# Controlled supramolecular assemblies of luminescent tridentate cyclometalated alkynylgold(III) amphiphiles in aqueous media

**DOI:** 10.3762/bjoc.22.88

**Published:** 2026-07-23

**Authors:** Kelvin Sze-Yim Cai, Brian Boyan Liu, Franco King-Chi Leung

**Affiliations:** 1 Department of Applied Biology and Chemical Technology, Research Institute of Future Food, The Hong Kong Polytechnic University, Hong Kong, China; 2 Centre for Eye and Vision Research, 17W Hong Kong Science Park, Hong Kong, China

**Keywords:** amphiphiles, gold(III) complex, luminescence, self-assembly, supramolecular chemistry

## Abstract

Gold(III) complexes and amphiphiles have been extensively investigated over a decade. Supramolecular assemblies of gold(III) amphiphiles in aqueous media exhibit high sensitivities to external stimulations for soft functional materials. Herein, we introduce a new molecular design of a tridentate cyclometalated gold(III) amphiphile (**GA**) with flexible molecular structure modifications. Counterion exchange with sodium tosylate induces notable luminescent enhancement and enables control over supramolecular assembly processes. This approach drives a supramolecular assembly transformation of **GA** from disordered nanosheets to well-ordered nanoribbons upon the additions of multiple equivalents of counterion, enabling a tunable pathway for controlled supramolecular transformation.

## Introduction

Supramolecular assemblies in natural systems, such as lipid bilayers and protein complexes, play essential roles in maintaining proper biological functions [1–3]. These natural structures have inspired developments of artificial supramolecular systems that offer structural diversity through delicate designed non-covalent interactions [4–7]. Amphiphilic molecules combine hydrophilic and hydrophobic groups and the intrinsic dual nature enables these molecules to spontaneously assemble into well-defined nanostructures, such as micelles, vesicles, and nanotubes [7–9]. Through implementations of various stimuli-responsive molecular motifs, amphiphiles can respond to external stimuli, such as light, pH, and heat [4,6,10–33], allowing them to be used in biomedical applications, such as, drug delivery and cancer immunotherapy [34–35]. An alternative molecular design approach is to incorporate organometallic complexes into amphiphile designs, thereby combining the advantageous functional properties of inorganic centers with the self-assembled structural properties of amphiphiles [36–40].

Gold(III) complexes have found wide applications for catalysis [41–44], optoelectronic materials [45–46], and bioconjugation methodologies [47–49], considering their unique luminescence properties and excellent aqueous stability. Amphiphilic gold(III) complexes were firstly reported by Che in 2016, who introduced polyethylene glycol (PEG)-functionalized gold(III) complexes to improve the biocompatibility and aqueous solubility [50]. Later, Che and co-workers developed charged cyclometalated gold(III) complexes, achieving kinetically controlled self-assembly in mixed solvents of acetonitrile and water through variation of counterions and its concentration [51]. Yam and co-workers have reported multiresponsive luminescent cationic gold(III) amphiphiles undergoing the self-assembly process in mixed organic solvents [52]. These reports demonstrate remarkable structural sensitivities to gold(III) amphiphiles molecular designs and the resulting nanostructural changes. In general, gold(III) complexes suffered from low luminescent properties due to the intrinsic low-energy *d–d* ligand field states and the enriched electrophilicity of the gold(III) metal center. The non-emissive low-lying *d*–*d* state of gold(III) complexes quenches the luminescent excited state through thermal equilibrium and energy transfer [53]. Cyclometalated gold(III) complexes incorporated with strong σ-donating alkynyl ligands, reported by Yam, exhibit enhanced luminescent properties under ambient conditions, which is attributed to the strong σ-donating alkynyl ligands [54–58].

Our research team has developed bidentate cyclometalated gold(III) amphiphiles featured with counterion-controlled reversible assembly in aqueous media [59–61]. However, the synthetic complexity and aqueous solubility issues of these molecular designs have hindered the functional applicability towards biomaterials. To overcome these limitations, we herein propose a new class of cyclometalated gold(III) amphiphiles **GA** featuring a tridentate C^N^C cyclometalated core connected to a strong σ-donating alkynyl ligand bearing a propargyl group linked to a terminal quaternary ammonium group through a nonyl linker. Compared with our earlier bidentate designs, this tridentate design provides greater structural rigidity and enhanced luminescence in aqueous media and ambident conditions. Furthermore, the synthetic pathway in this work offers significantly increased accessibility and flexibility of the molecular structural modifications. The incorporation of the propargyl–nonyl–quaternary ammonium motif not only enhances aqueous solubility but also enables fine adjustments of the packing parameter of **GA** through counterion exchange, thereby achieving a controllable transformation of supramolecular nanostructures. With meticulous investigations of self-assembly processes, photophysical properties, and counterion-dependent structural transformations of **GA**, a new molecular framework for counterion-controlled luminescent soft materials can be established towards biomedical and biomaterial applications.

## Results and Discussion

### Design and synthesis of **GA**

**Scheme 1:**
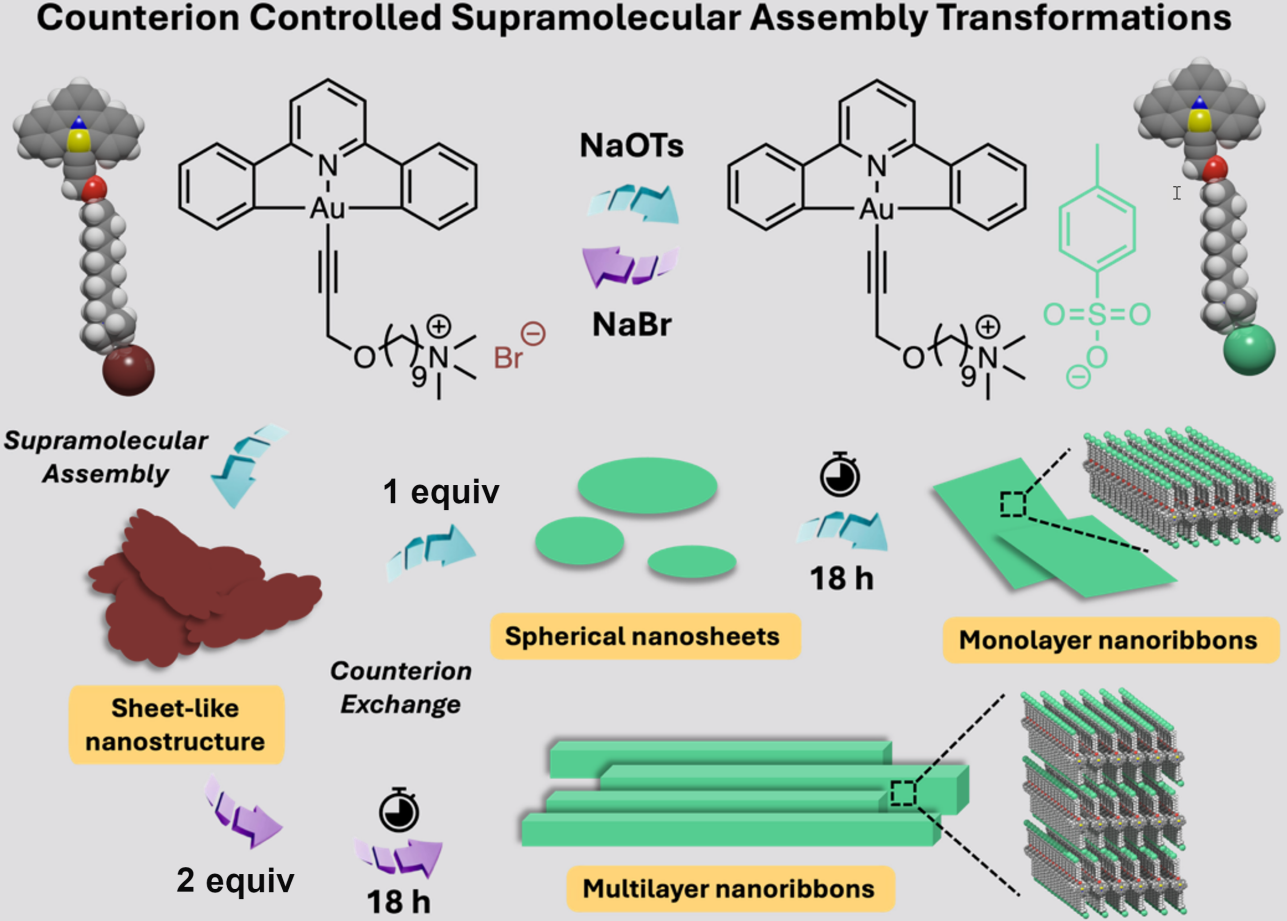
Schematic illustration of the design of **GA** and the corresponding supramolecular assembly transformations.

**Scheme 2:**
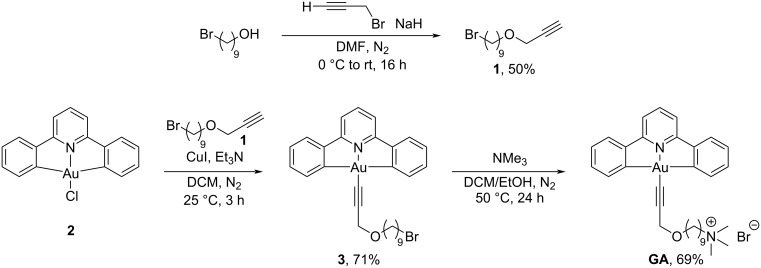
Synthetic pathway of **GA**.

The gold amphiphile was designed with a tridentate C^N^C cyclometalated gold(III) complex core, which is functionalized with a σ-donating alkynyl ligand. A propargyl group was connected by a nonyl-linker with a quaternary ammonium ion as the charged end group (Scheme 1). The synthetic route of **GA** is shown in Scheme 2. Compound **1** was obtained by the substitution of propargyl bromide with 9-bromo-1-nonanol. Based on the previously reported procedure, the cyclometalated gold(III) complex **2** was synthesized and further reacted with compound **1** in the presence of triethylamine and a catalytic amount of copper iodide to afford the amphiphile precursor **3**. The nucleophilic substitution of compound **3** with trimethylamine enabled to afford **GA**. The chemical structures of all newly synthesized compounds and **GA** were unambiguously characterized by ^1^H , ^13^C NMR spectroscopy and high-resolution mass spectrometry (HRMS) (Supporting Information File 1, Figures S8–S13).

### Photophysical properties and assembly of **GA**

**Figure 1:**
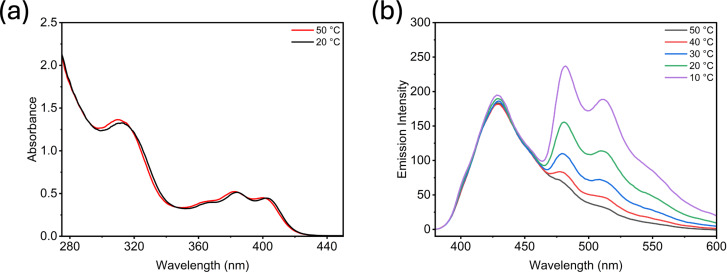
(a) UV–vis absorption spectra of **GA** (200 μM) in MQ water heated to 50.0 °C (red line) and cooled to 20.0 °C (black line) at a rate of 1.0 °C/min. (b) Emission spectra of **GA** (200 μM) in MQ water upon decreasing the temperature from 50.0 °C (black line) to 10.0 °C (purple line).

A freshly prepared aqueous solution of **GA** (13.4 mM, 1.0 wt %) was annealed at 50.0 °C for 5 min and cooled to room temperature as the stock solution. The stock solution of **GA** was subsequently diluted into a range of concentrations from 0.01 to 1.0 mM, in estimating the critical aggregation concentration (CAC) by static light scattering measurements (SLS). The CAC of **GA** was determined as 10 µM (Supporting Information File 1, Figure S1), representing plausible formation of **GA** supramolecular assemblies in the aqueous environment. A diluted solution of **GA** (200 µM) was further thermally annealed by heating to 50.0 °C for 5 min and slow cooling to 20.0 °C at a rate of 1.0 °C/min. The UV–vis absorption spectrum of the solution shows an intense absorption band at 304–318 nm and a moderately intense vibronic-structured absorption band at 360–405 nm at 20.0 °C (Figure 1a, black line). The vibrational progressional spacings of 1300–1310 cm^−1^ in the lower energy band represent the skeletal vibrational frequencies of the cyclometalated C^N^C ligand. In considering similar absorption bands observed in the cyclometalated gold(III) complex **2** [62] and the related alkynylgold(III) complexes [54–56], the vibronic-structured absorption band at 360–405 nm is revealed as insensitive to the structurally modified alkynyl ligands, which is generally assigned as a metal-perturbed intraligand (IL) π–π* transition of the C^N^C ligand. A subtle increase in absorbance with a hypochromic-shift of 3 nm was observed upon heating the solution to 50.0 °C (Figure 1a, red line), which was reversed upon cooling to 20.0 °C. This thermal annealing process is fully reversible with clear isosbestic points, suggesting that the supramolecular assembly of **GA** in aqueous media is under thermodynamic control without observable degradation. A methanol solution of **GA** (200 µM) shows no signs of spectral shift and is reversible upon annealing (Supporting Information File 1, Figure S2a), indicating the absence of supramolecular aggregation in an organic environment and the thermal stability of **GA** in both organic and aqueous media.

Upon excitation a methanol solution of **GA** (200 µM) with 365 nm, a broad and structureless emission band at 428 nm is observed at 20.0 °C (Supporting Information File 1, Figure S2b, green line). In contrast, an aqueous solution of **GA** (200 µM) exhibits an additional vibronic-structured emission band at 465–540 nm, and the intensity of the emission band at 429 nm (Figure 1b, green-line) is lower than that of **GA** in organic media (Supporting Information File 1, Figure S2b, green line) at 20.0 °C. Given that a dichloromethane solution of cyclometalated gold(III) complex **2** (200 µM) shows significantly lowered emission intensity at 430 nm (Supporting Information File 1, Figure S3), the emission spectral results suggested that the luminescent properties of **GA** have been greatly improved through the coupling with a σ-donor to gold(III) complex. Upon progressively heating the aqueous solution of **GA** from 10.0 °C to 50.0 °C, the resulting emission intensity of the vibronic-structured emission band was gradually decreased along with the subtly decreased structureless emission band at 428 nm (Figure 1b), possibly due to emissions from the **GA** assemblies.

Upon increasing the water content in methanol, the UV–vis absorption spectra of **GA** show a progressive increase in absorbance of the IL band tail at 420 nm (Supporting Information File 1, Figure S4a and S4b), suggesting the formation of aggregates. The emission spectra of **GA** show a reduction in intensity of the structureless emission band at 429 nm with increased emission of the lower-energy vibronic-structured emission band at 465–540 nm upon water content increased from 0% to 99% (Supporting Information File 1, Figure S4c and S4d). The obtained results demonstrated a clear redistribution of emission intensity due to aggregation of **GA** in aqueous media. The rigid molecular packed structures of **GA** in the aggregated form restrict intramolecular vibrations of the C^N^C ligand and suppress non-radiative decay pathways to activate the lower-energy emission. An essentially identical emission spectrum was observed for the methanol solution of **GA** at 20.0 °C (Supporting Information File 1, Figure S2b, green line) to that of an aqueous solution of **GA** at 50 °C (Figure 1b, black line). This can be illustrated by the vibronic-structured emission band quenched at increased temperature and in the organic medium due to possible non-radiative decay pathways. These results further confirmed that the vibronic-structure emission band can be induced upon **GA** assembly, which shows possible correlations with temperature and water content.

### Counterion-controlled supramolecular assembly transformations of **GA**

**Figure 2:**
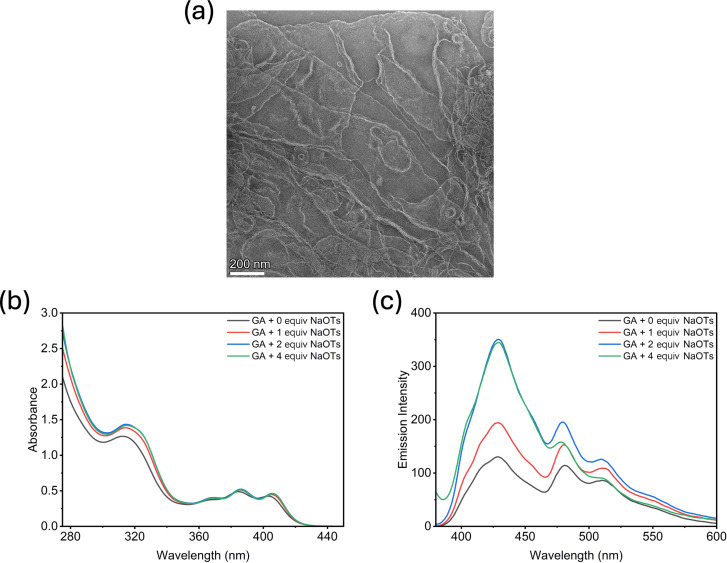
(a) TEM image of a thermally annealed aqueous solution of **GA** (2.68 mM). (b) UV–vis absorption spectra and (c) emission spectra of **GA** (200 μM) in Milli-Q water at 20.0 °C after addition of sodium tosylate (1.0, 2.0, and 4.0 equiv).

Negative-stained transmission electron microscopy (TEM) was used to analyze the assembled nanostructures of **GA**. TEM images of a thermally annealed aqueous solution of **GA** (2.68 mM, 0.2 wt %) reveal sheet-like nanostructures with widths ranging from hundreds of nanometers to micrometers (Figure 2a). To a thermally annealed aqueous solution of **GA** (200 μM) were added varying equivalents of an aqueous solution of sodium tosylate (1.0, 2.0, and 4.0 equiv), and subsequently heated to 50.0 °C and slowly cooled to 20.0 °C at a rate of 1.0 °C/min. The UV–vis absorption spectra of the solution show a progressive bathochromic shift with increasing sodium tosylate equivalents (Figure 2b). The presence of isosbestic points at 359 nm, 375 nm, 381 nm, 395 nm, and 401 nm within the vibronic-structured absorption band indicates a selective counterion exchange process, where the organic tosylate anion replaces the inorganic bromide anion. The corresponding emission spectra of the solution show a notable enhancement in the overall intensity upon the addition of 1.0–2.0 equiv of sodium tosylate (Figure 2c red line and blue line). However, the vibronic-structured emission band diminished upon the addition of 4.0 equiv of sodium tosylate (Figure 2c, green line).

**Figure 3:**
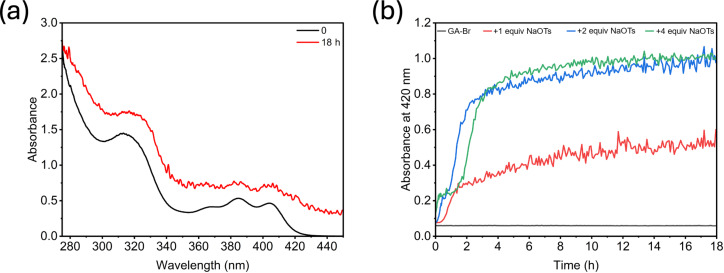
(a) Time-dependent UV–vis adsorption spectra of **GA** (200 μM) with addition of 1.0 equiv of sodium tosylate in Milli-Q water at 20.0 °C. (b) Time-dependent absorbance changes of **GA** at 420 nm with and without the addition of sodium tosylate (1, 2, or 4 equiv).

The time-dependent UV–vis adsorption spectra of the annealed solution of **GA** without the addition of sodium tosylate show no significant changes after 18 h at 20.0 °C (Supporting Information File 1, Figure S5a). In contrast, the absorbance of the annealed solution of **GA** with 1.0, 2.0 and 4.0 equiv of sodium tosylate gradually increased after 18 hours of aging (Figure 3a, and Figures S5b and S5c in Supporting Information File 1), indicating the formation of a thermodynamically driven assembly. A clear lag time for **GA** with tosylate (1.0 equiv) suggests the presence of a metastable assembly state (Figure 3b, red line). The spontaneous assembly process is suppressed due to stronger intermolecular interactions between the **GA** cation end group and the tosylate anion than with the bromide anion [51,63]. Increasing the concentration of sodium tosylate from 1.0 to 4.0 equiv progressively reduces the lag time and accelerates the assembly process (Figure 3b). Notably, the final absorbance intensity of **GA** with tosylate (2.0 and 4.0 equiv) is approximately twice that of **GA** with tosylate (1.0 equiv), suggesting a significant difference in the density or size of the thermodynamically driven assemblies formed (Figure 3b).

**Figure 4:**
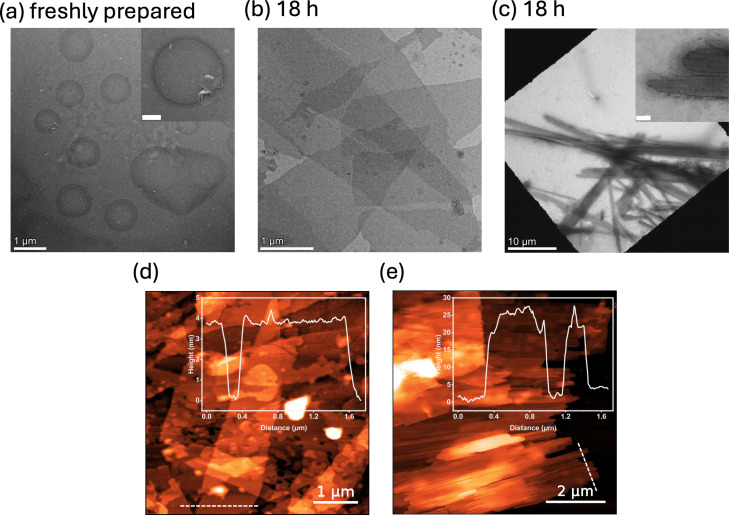
Time-dependent TEM changes of the thermally annealed **GA** solution (2.68 mM) with the addition of 1.0 equiv of sodium tosylate (a) freshly prepared (inset: higher magnification of spherical nanosheets, scale bar: 200 nm) and (b) after 18 h. (c) TEM image of the thermally annealed **GA** solution (2.68 mM) with the addition of 2.0 equiv of sodium tosylate obtained at 18 h (inset: higher magnification of long nanoribbons, scale bar: 500 nm). AFM images of the aged **GA** solution (2.68 mM) with the addition of (d) 1.0 equiv and (e) 2.0 equiv of sodium tosylate (inset: height profile along the indicated line).

A freshly prepared thermally annealed aqueous solution of **GA** (2.68 mM, 0.2 wt %) with tosylate (1.0 equiv) was imaged with spherical nanosheets with diameters ranging from ≈200 nm to 2 µm (Figure 4a). After being aged for 18 h, the spherical nanosheets of **GA** have evolved into stacked nanoribbons (Figure 4b). The time-dependent transitions indicate a kinetically controlled assembly process, in which the initial metastable assemblies transform into their thermodynamically driven assemblies. Upon addition of 2.0 equiv of tosylate and aging for 18 h, long nanoribbons were observed (Figure 4c). In contrast, irregular assemblies were observed in an aged **GA** solution with 4.0 equiv of tosylate (Supporting Information File 1, Figure S6), possibly due to the disruption of ordered assemblies by supersaturated ions. Atomic force microscopy (AFM) was performed to further confirm the assembled nanostructure of the aged **GA** solution (2.68 mM, 0.2 wt %) with tosylate (1.0 and 2.0 equiv). Essentially identical nanoribbon structures were found with a uniform height of 3.8 nm and 25 nm (Figure 4d and 4e). Given that the molecular length of **GA** is estimated to be approximately 2.4 nm, it is assumed that the nanoribbons of **GA** with 1.0 equiv of tosylate are monolayers in which two **GA** molecules exhibit head-to-tail stacking, while **GA** with 2.0 equiv of tosylate forms multilayer nanoribbons. These results demonstrate that counterion exchange from bromide to tosylate enhances the packing parameter of **GA**, resulting in a transition from less ordered nanosheets to well-ordered nanoribbons. In considering the significant influence of counterions, to the solution of **GA** (2.68 mM, 0.2 wt %) with tosylate (1.0 and 2.0 equiv) were added the corresponding equivalents of sodium bromide followed by further thermal annealing. The TEM images of the resulting solution revealed the reverse supramolecular transformation from regular nanoribbons to sheet-like nanostructures (Supporting Information File 1, Figure S7).

The distinct assembly outcomes observed with bromide and tosylate can be rationalized by considering their effects on the molecular packing parameter. As a small, highly hydrated inorganic anion, bromide maintains a larger effective head group area, resulting in a packing parameter that favours the formation of relatively flat but disordered lamellar nanosheets. In contrast, tosylate, being a larger and more hydrophobic organic anion with an aromatic moiety, reduces the effective head group area through lower hydration and additional π–π stacking and hydrophobic interactions. This shift in the packing parameter promotes anisotropic growth, enabling the transformation from disordered nanosheets into well-ordered monolayer nanoribbons and eventually multilayer nanoribbons with increasing tosylate concentration. These findings highlight the critical role of counterion nature in directing the hierarchical organization of gold(III) amphiphiles in aqueous media.

## Conclusion

A tridentate cyclometalated gold(III) amphiphile, functionalized with a σ-donating alkynyl ligand, was designed and synthesized. This structural modification significantly enhanced its luminescent properties in aqueous media. Supramolecular assembled nanostructures of **GA** were imaged and confirmed by TEM and AFM. The addition of sodium tosylate, enabled a thermodynamically driven assembly process, leading to supramolecular assembly transformations from sheet-like nanostructures to spherical nanosheets, and to stacked nanoribbons throughout an aging process. Well-defined monolayered and multilayered nanoribbons were observed by varying concentrations of tosylate ions. The reverse supramolecular transformation was enabled in the current gold(III) amphiphilic design by the addition of bromide ions. The current system of a tridentate cyclometalated gold(III) amphiphile lays the foundation for developing next generations of controlled supramolecular assembly of gold(III) amphiphiles.

## Experimental

**Materials:** All commercial reagents were purchased from Acros Organics, Aladdin, Bidepharm, Dieckmann, Macklin and Tokyo Chemical Industry Co. Ltd, and were used as received unless otherwise specified. All reactions were performed under nitrogen unless otherwise specified. Analytical thin-layer chromatography (TLC) was performed with Macherey-Nagel Silica gel 60 UV_254_ aluminum plates and visualization was accomplished by UV light (254 nm) or staining with phosphomolybdic acid (PMA) followed by heating. Flash column chromatography was performed using Macherey-Nagel Silica gel 60 (230–400 mesh). Deuterated solvents were purchased from Cambridge Isotope Laboratories Inc.

**Compound 1:** Synthesis according to the modification of a reported procedure [64]. A mixture of 9-bromo-1-nonanol (482 mg, 2.16 mmol) and sodium hydride (100 mg, 4.17 mmol) in dimethylformamide (5 mL) was stirred at 0 °C for 15 min. Propargyl bromide (0.2 mL, 2.59 mmol) was added dropwise to the mixture solution and stirred at room temperature for 16 h. The resulting mixture was washed with brine (25 mL × 1), water (25 mL × 2), and brine (25 mL × 1). The organic layer was dried over sodium sulfate. The solvent was evaporated under vacuum and the residue subjected to column chromatography on silica gel (hexane/ethyl acetate 10:1 (v/v, *R*_f_ = 0.5) to afford compound **1** as pale-yellow liquid (284 mg, 1.09 mmol) in 50% yield. ^1^H NMR (600 MHz, CDCl_3_) δ 4.08 (s, 2H), 3.46 (t, *J* = 6.6 Hz, 2H), 3.36 (t, *J* = 7.0 Hz, 2H), 2.39 (s, 1H), 1.80 (q, *J* = 7.3 Hz, 2H), 1.54 (t, *J* = 6.4 Hz, 2H), 1.37 (t, *J* = 7.4 Hz, 2H), 1.31–1.25 (m, 8H); HRMS–ESI^+^ (*m*/*z*): [M + H]^+^ calcd for C_12_H_22_BrO, 261.0849; found, 261.0848.

**Compound 2:** Synthesis according to the modification of a reported procedure [62]. ^1^H NMR (600 MHz, CD_3_SOCD_3_) δ 8.19 (t, *J* = 8.0 Hz, 1H), 8.01–7.97 (m, 2H), 7.89 (d, *J* = 7.7 Hz, 2H), 7.70 (d, *J* = 7.2 Hz, 2H), 7.43 (t, *J* = 7.3 Hz, 2H), 7.32 (t, *J* = 7.6 Hz, 2H).

**Compound 3:** A mixture of compound **1** (87 mg, 0.33 mmol) and compound **2** (103 mg, 0.22 mmol) in the presence of a catalytic amount of copper(I) iodide (4 mg, 0.02 mmol) in triethylamine (1 mL) and dichloromethane (20 mL) was stirred at room temperature for 3 h. The resulting mixture was dried under vacuum and subjected to column chromatography on silica gel (hexane/dichloromethane 1:2 (v/v, *R*_f_ = 0.5) to afford compound **3** as yellow solid (109 mg, 0.16 mmol) in 71% yield. ^1^H NMR (400 MHz, CDCl_3_) δ 7.98 (d, *J* = 7.3 Hz, 2H), 7.83 (t, *J* = 7.9 Hz, 1H), 7.51 (d, *J* = 7.7 Hz, 2H), 7.43 (d, *J* = 7.9 Hz, 2H), 7.35 (t, *J* = 6.7 Hz, 2H), 7.22 (t, *J* = 7.6 Hz, 2H), 4.47 (s, 2H), 3.73 (t, *J* = 6.7 Hz, 2H), 3.38 (t, *J* = 6.9 Hz, 2H), 1.83 (p, *J* = 7.0 Hz, 2H), 1.76–1.64 (m, 2H), 1.42 (dt, *J* = 14.4, 7.6 Hz, 4H), 1.32 (q, *J* = 5.6 Hz, 6H); ^13^C NMR (101 MHz, CDCl_3_) δ 166.85, 165.10, 149.11, 142.35, 136.77, 132.06, 126.85, 125.27, 116.85, 96.68, 86.21, 77.36, 69.56, 60.35, 34.25, 33.00, 29.88, 29.59, 28.89, 28.34, 26.46; HRMS–ESI^+^ (*m*/*z*): [M + H]^+^ calcd for C_29_H_32_AuBrNO, 686.1328; found, 686.1319.

**GA:** A mixture of compound **3** (92 mg, 0.13 mmol) and 20% trimethylamine in ethanol (1 mL) in dichloromethane (2 mL) was heated at 50 °C for 24 h in a Schlenk tube. The resulting mixture was cooled to room temperature and precipitated with dichloromethane and diethyl ether. The precipitate was filtered off and was washed with hexane to afford **GA** as pale-yellow solid (69 mg, 0.09 mmol) in 69% yield. ^1^H NMR (600 MHz, MeOD) δ 8.02–7.97 (m, 1H), 7.86 (d, *J* = 7.7 Hz, 2H), 7.70 (t, *J* = 7.9 Hz, 4H), 7.31 (t, *J* = 6.6 Hz, 2H), 7.27–7.21 (m, 2H), 4.40 (s, 2H), 3.73 (t, *J* = 6.3 Hz, 2H), 3.26 (s, 2H), 3.08 (s, 9H), 1.76 (q, *J* = 8.0 Hz, 2H), 1.69 (q, *J* = 7.1 Hz, 2H), 1.48 (d, *J* = 7.1 Hz, 2H), 1.38 (d, *J* = 25.2 Hz, 8H); ^13^C NMR (151 MHz, MeOD) δ 167.70, 166.23, 150.82, 144.43, 137.36, 132.60, 128.01, 126.67, 118.53, 70.40, 60.99, 53.43, 30.61, 30.40, 30.28, 30.06, 27.29, 23.88; HRMS–ESI^+^ (*m*/*z*): [M – Br]^+^ calcd for C_32_H_40_AuN_2_O, 665.2801; found, 665.2846.

**Preparation of aqueous sample: GA** (13.4 mM, 1.0 wt %) was dissolved in fresh Milli-Q water (MQ water). The solution was heated at 50.0 °C for 5 min, then slowly cooled to 20.0 °C at a rate of 1.0 °C/min to form assembled structures.

**UV–vis spectroscopy:** UV–vis measurements were performed on an Agilent Cary 60 UV–vis spectrophotometer with a 1 cm path length quartz cuvette. A Luma 40/8453 temperature-controlled cuvette holder with four optical ports was mounted in the sample compartment of the Agilent Cary 60 UV–vis spectrophotometer. Time-dependent UV–vis adsorption measurements were performed using a small stirring bar (3 × 5 mm) at a constant stirring speed of 1200 rpm. Slits were installed on the cuvette holder to prevent the stirring bar from interfering with the optical path to the spectrometer. Measurements of all samples were carried out at 20.0 °C unless otherwise specified.

**Fluorescence spectroscopy:** Fluorescence measurements were performed on an Agilent G9800A Cary Eclipse fluorescence spectrophotometer with a 1 cm path length quartz cuvette. A Luma 40/8453 temperature-controlled cuvette holder with four optical ports was mounted in the sample compartment of the Agilent G9800A Cary Eclipse fluorescence spectrophotometer. Measurements of all samples were carried out at 20.0 °C unless otherwise specified.

**Static light scattering (SLS):** Static light scattering intensities of each sample were determined on a Wyatt Technology DynaPro NanoStar. The scattering intensities were recorded as a parameter for assembly size, given that the objects in solution are anisotropic and the models used by Wyatt software are fitting for spherical objects. The critical aggregation concentration (CAC) of **GA** was determined by the scattering intensities of the solutions of **GA** (concentration: 0.01 to 1.0 mM) at 20.0 °C. The scattering rate was normalized by the concentration of the solution to yield the molar scattering intensity (M counts s^−1^ M^−1^). Ten replications were performed, and the data was averaged to show the molar scattering intensity and corresponding standard deviation.

**Treatment of GA with sodium tosylate for counter anion-controlled supramolecular transformation:** To a thermally annealed aqueous solution of **GA** (13.4 mM) was added an aqueous solution of sodium tosylate (1.0, 2.0, and 4.0 equiv). The obtained solution was heated at 50.0 °C for 5 min, and slowly cooled to 20.0 °C at a rate of 1.0 °C/min.

**Transmission electron microscopy (TEM):** TEM samples were prepared by depositing sample solutions (0.2 wt %, 10.0 μL) onto a carbon grid (Micro to Nano, EMR Carbon support film on copper, 400 square mesh) for 30 s. The sample solution was removed by blotting and UranyLess EM stain solution (Electron Microscopy Science, 5.0 μL) was directly deposited onto the grid for 15 s, and the stain was removed by blotting. Grids were observed in a ThermoFisher Talos L120C transmission electron microscope at an accelerating voltage of 120 kV. TEM images were captured on a Ceta 16M CCD camera.

**Atomic force microscopy (AFM):** The atomic force microscopy was performed on a JPK NanoWizard V BioScience AFM (Bruker) equipped on Nikon ECLIPSE Ti-2 microscope with a cantilever (RFESPG-75, Bruker). The measurement of the assembled morphology was conducted in air with the Peakforce Tapping mode. The sample solution (0.2 wt %, 5.0 μL) was dropped onto a freshly cleaved mica plate surface and spin-coated to remove excess solvent.

## Supporting Information

File 1 Experimental details, supporting figures, and copies of spectra.

## Data Availability

All data that supports the findings of this study is available in the published article and/or the supporting information of this article.
