# Supplementary material for: Controlled supramolecular assemblies of luminescent tridentate cyclometalated alkynylgold(III) amphiphiles in aqueous media
Source: Beilstein J Org Chem. 2026 Jul 23;22:1097–106. doi: 10.3762/bjoc.22.88 (PMC13402995; doi:10.3762/bjoc.22.88)
Supplement: File 1 — Experimental details, supporting figures, and copies of spectra. [file Beilstein_J_Org_Chem-22-1097-s001.pdf]

BEILSTEIN JOURNAL OF ORGANIC CHEMISTRY

# Supporting Information

for

# Controlled supramolecular assemblies of luminescent tridentate cyclometalated alkynylgold(III) amphiphiles in aqueous media

Kelvin Sze-Yim Cai, Brian Boyan Liu and Franco King-Chi Leung

*Beilstein J. Org. Chem.* **2026,** *22,* 1097–1106. doi:10.3762/bjoc.22.88

# Experimental details, supporting figures, and copies of spectra

License and Terms: This is a supporting information file under the terms of the Creative Commons Attribution License (https://creativecommons.org/licenses/by/4.0). Please note that the reuse, redistribution and reproduction in particular requires that the author(s) and source are credited and that individual graphics may be subject to special legal provisions.
The license is subject to the *Beilstein Journal of Organic Chemistry* terms and conditions: (https://www.beilstein-journals.org/bjoc/terms)

## Table of contents

1. General ........................................................................................................S2

2. Supporting figures ..........................................................................................S3

3. Compound characterization ........................................................................S10

## 1. General

NMR spectra were recorded at 20.0 °C on Bruker AVANCE III 600 NMR spectrometer ($^{1}H$: 600 MHz, $^{13}C$: 151 MHz) or Bruker AVANCE III 400 NMR spectrometer ($^{1}H$: 400 MHz, $^{13}C$: 101 MHz). Chemical shifts (δ) are expressed relative to the resonances of the residual nondeuterated solvent for $^{1}H$ [$CDCl_3$: $^{1}H(\delta)$ = 7.26 ppm, $CD_3OD$: $^{1}H(\delta)$ = 3.31 ppm, $CD_3SOCD_3$: $^{1}H(\delta)$ = 2.50 ppm] and $^{13}C$ [$CDCl_3$: $^{13}C(\delta)$ = 78.0 ppm, $CD_3OD$: $^{13}C(\delta)$ = 49.15 ppm]. Absolute values of the coupling constants are given in hertz (Hz), regardless of their sign. Multiplicities are abbreviated as singlet (s), doublet (d), doublet of doublets (dd), triplet (t), triplet of doublets (td), quartet (q), multiplet (m), and broad (br). High-resolution mass spectrometry (HRMS) was performed on an Agilent 6540 UHD Accurate-Mass Q-TOF LC/MS system with ESI ionization.

## 2. Supporting figures

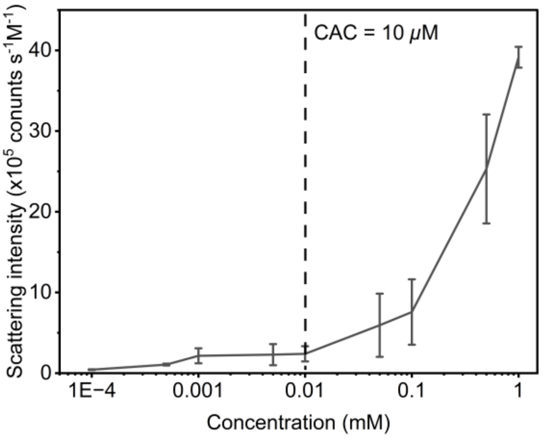


**Figure S1:** The critical aggregation concentration of aqueous solution of **GA**.

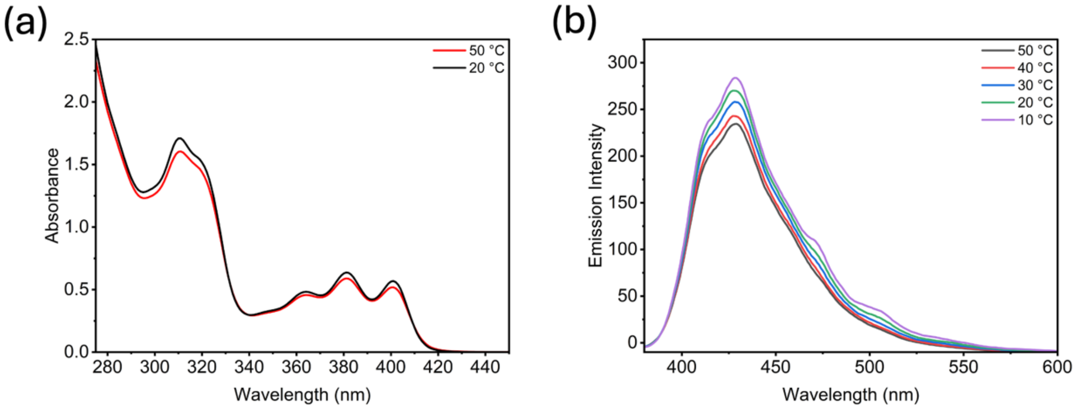


**Figure S2:** (a) UV–vis absorption spectra of **GA** (200 μM) in methanol was cooled from 50.0 °C (red line) to 20.0 °C (black line) at a rate of 1.0 °C/min. (b) Emission spectra of **GA** (200 μM) in methanol upon decreasing temperature from 50.0 °C to 10.0 °C.

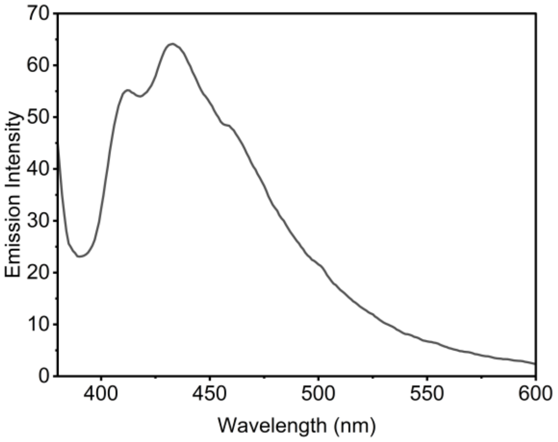


**Figure S3:** Emission spectrum of the cyclometalated gold(III) complex **2** in dichloromethane (200 μM).

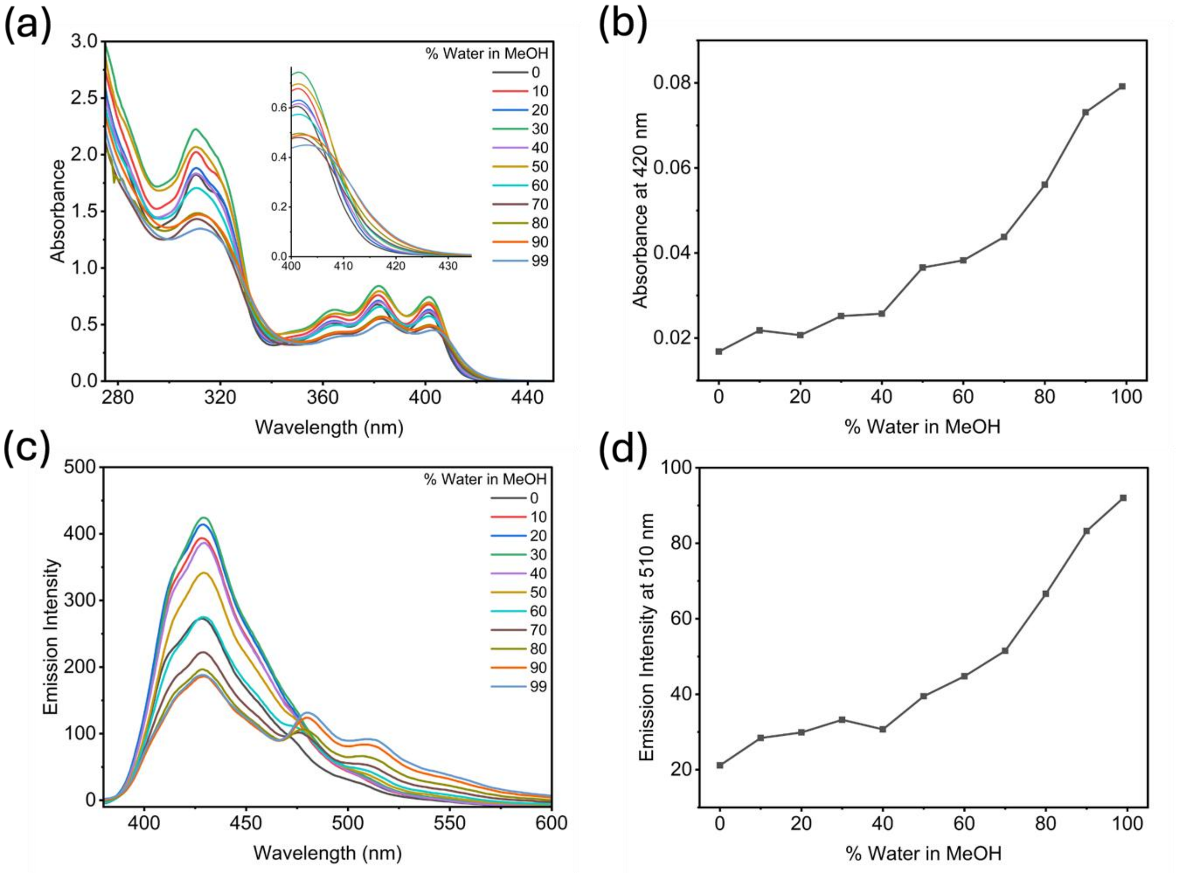


**Figure S4:** (a) UV–vis absorption spectra of **GA** (200 μM) in methanol at 20.0 °C upon increasing water content. (b) A plot of absorbance at 420 nm against water fraction in methanol. (c) Emission spectra of **GA** (200 μM) at 20.0 °C upon increasing water content. (d) A plot of emission at 430 nm and 510 nm against water fraction in methanol.

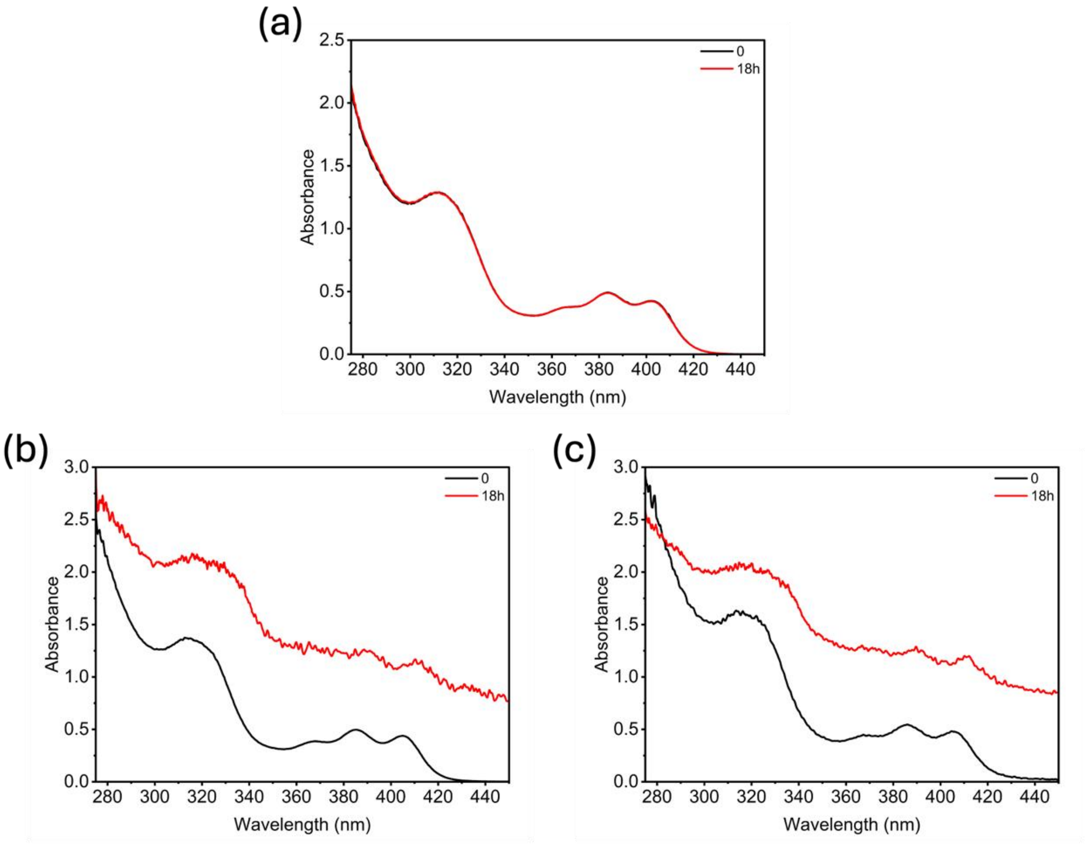


**Figure S5:** Time-dependent UV–vis adsorption spectra of **GA** (200 μM) (a) without the addition of sodium tosylate, with the addition of (b) 2.0 equiv and (c) 4.0 equiv of sodium tosylate in MQ water at 20.0 °C.

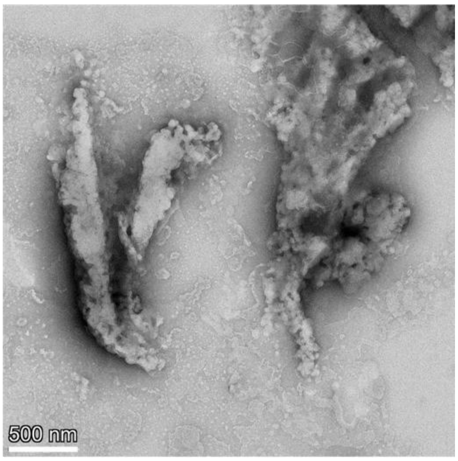


**Figure S6:** TEM image of thermally annealed **GA** solution (2.68 mM) with the addition of 4.0 equiv of sodium tosylate obtained at 18 h.

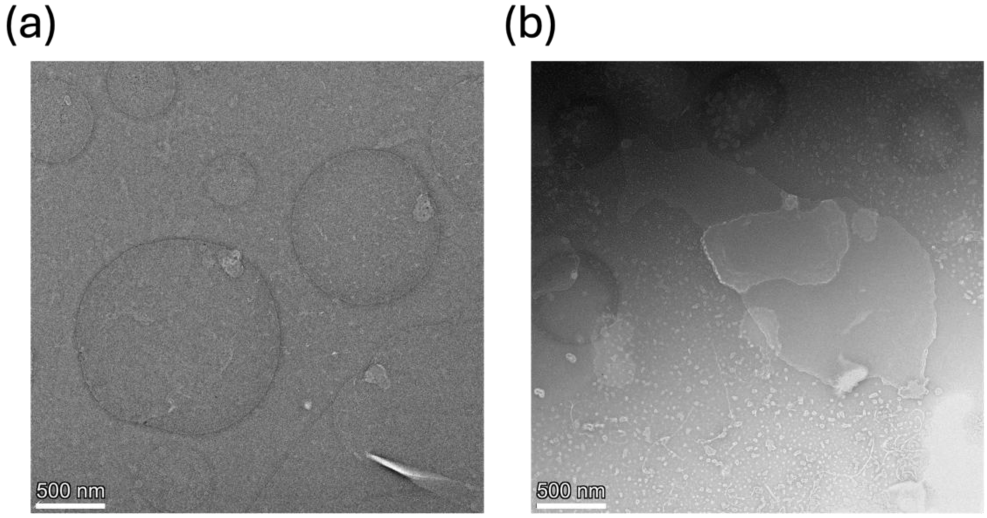


**Figure S7:** TEM images of thermally annealed solution of **GA** (2.68 mM) after addition of (a) 1.0 equiv and (b) 2.0 equiv of sodium bromide as further counterion exchange.

## 3. Compound characterization

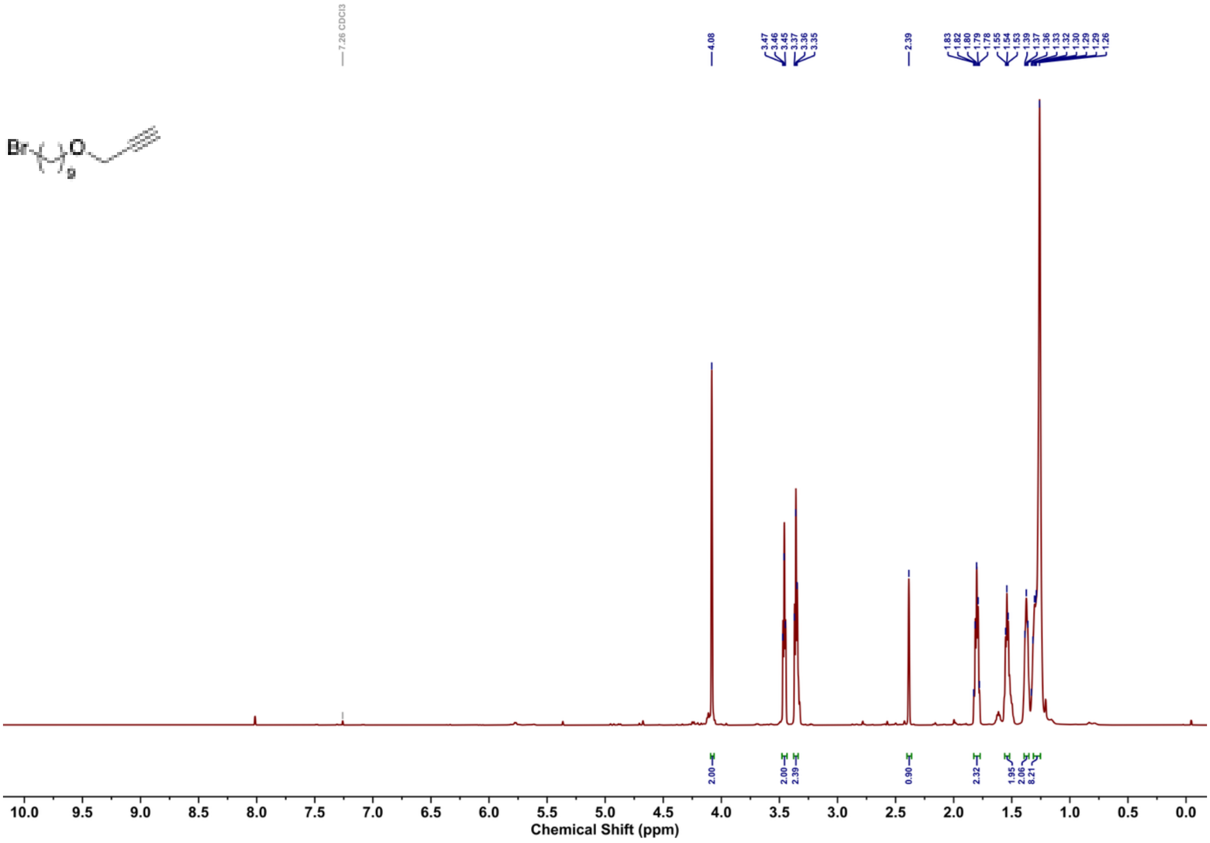


**Figure S8:** $^{1}$H NMR spectrum (600 MHz) of compound **1** in $CDCl_3$ at 20.0 °C.

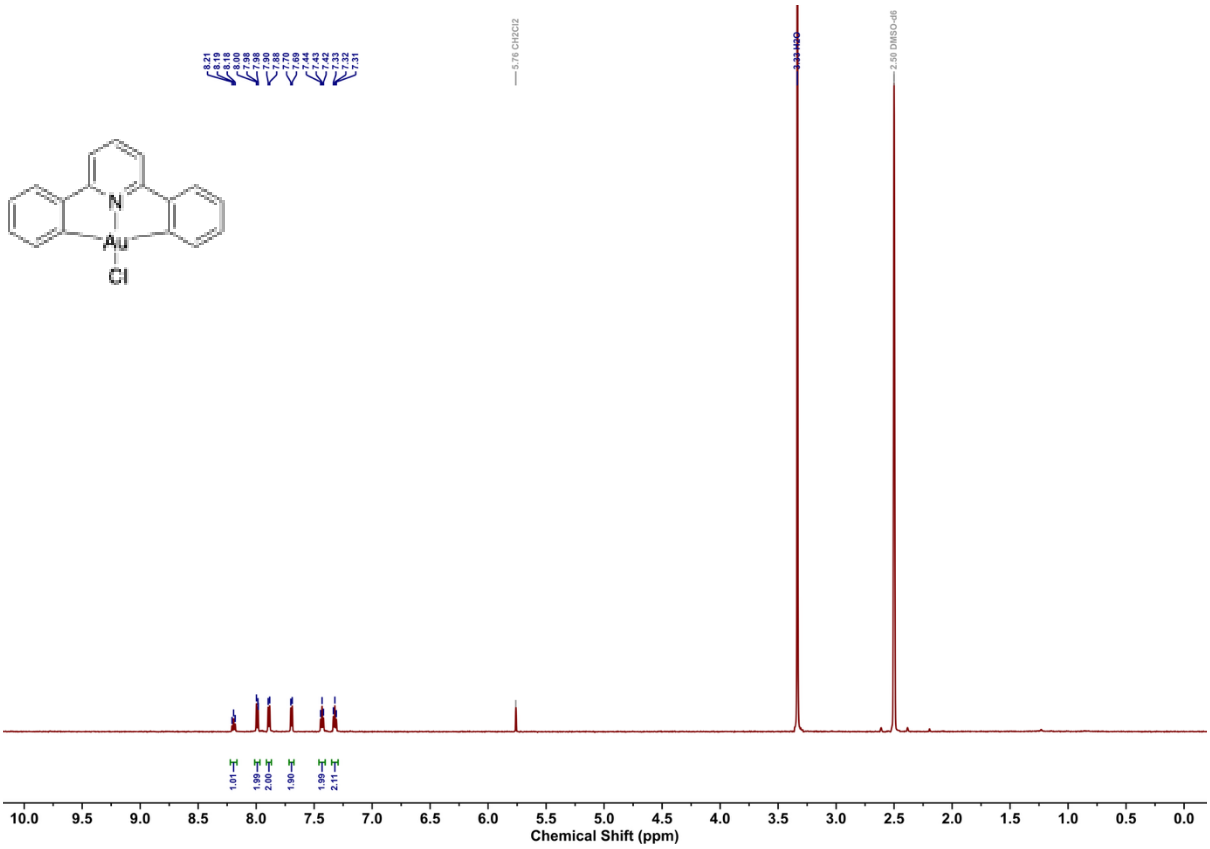


**Figure S9:** $^{1}H$ NMR spectrum (600 MHz) of compound **2** in $CD_3SOCD_3$ at 20.0 °C.

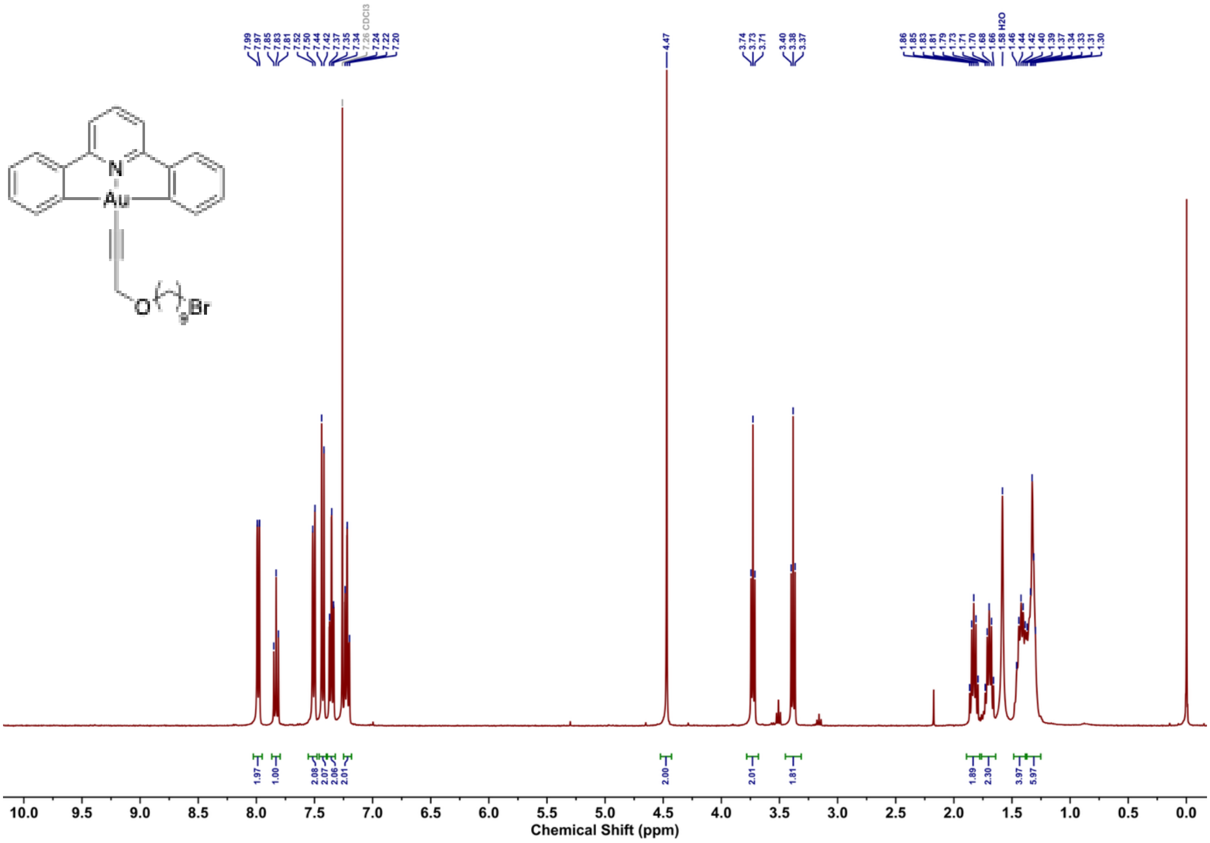


**Figure S10:** $^{1}$H NMR spectrum (400 MHz) of compound **3** in $CDCl_3$ at 20.0 °C.

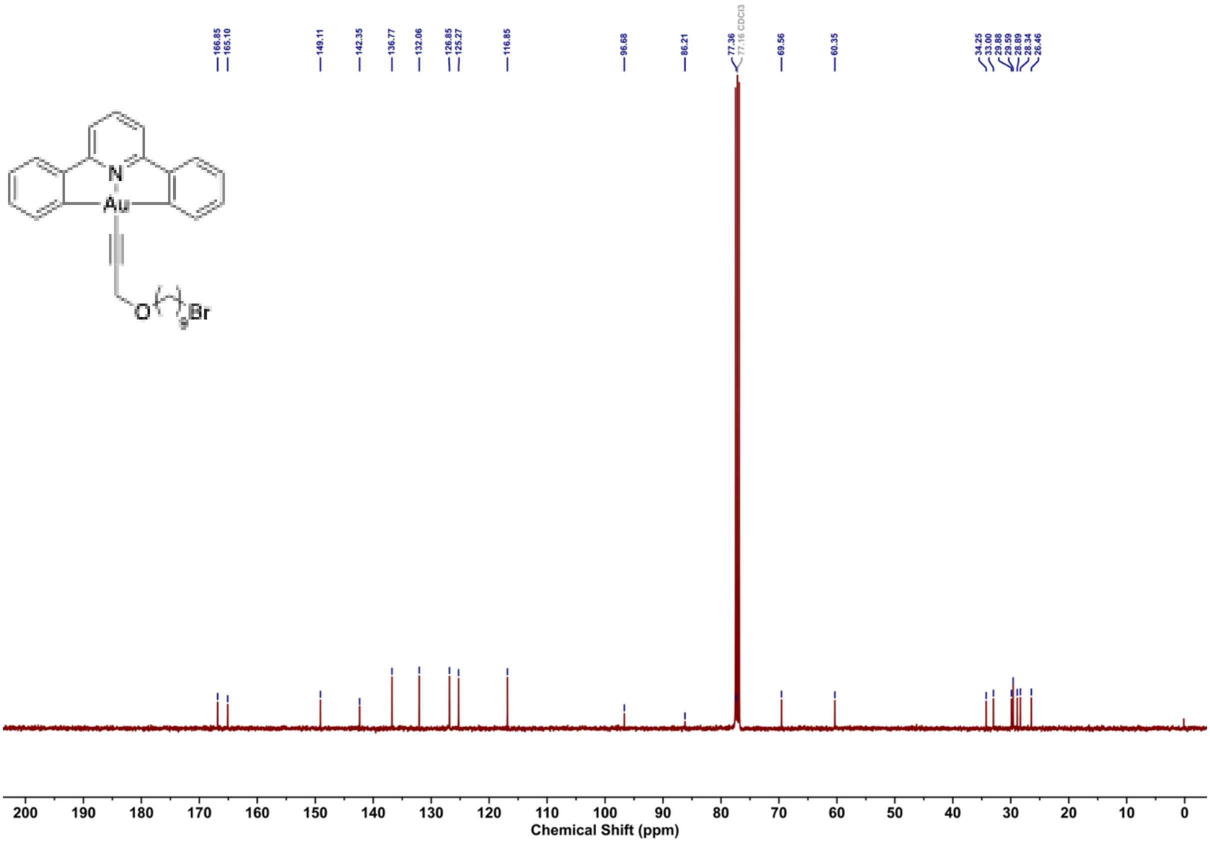


**Figure S11:** $^{13}C$ NMR spectrum (101 MHz) of compound **3** in $CDCl_3$ at 20.0 °C.

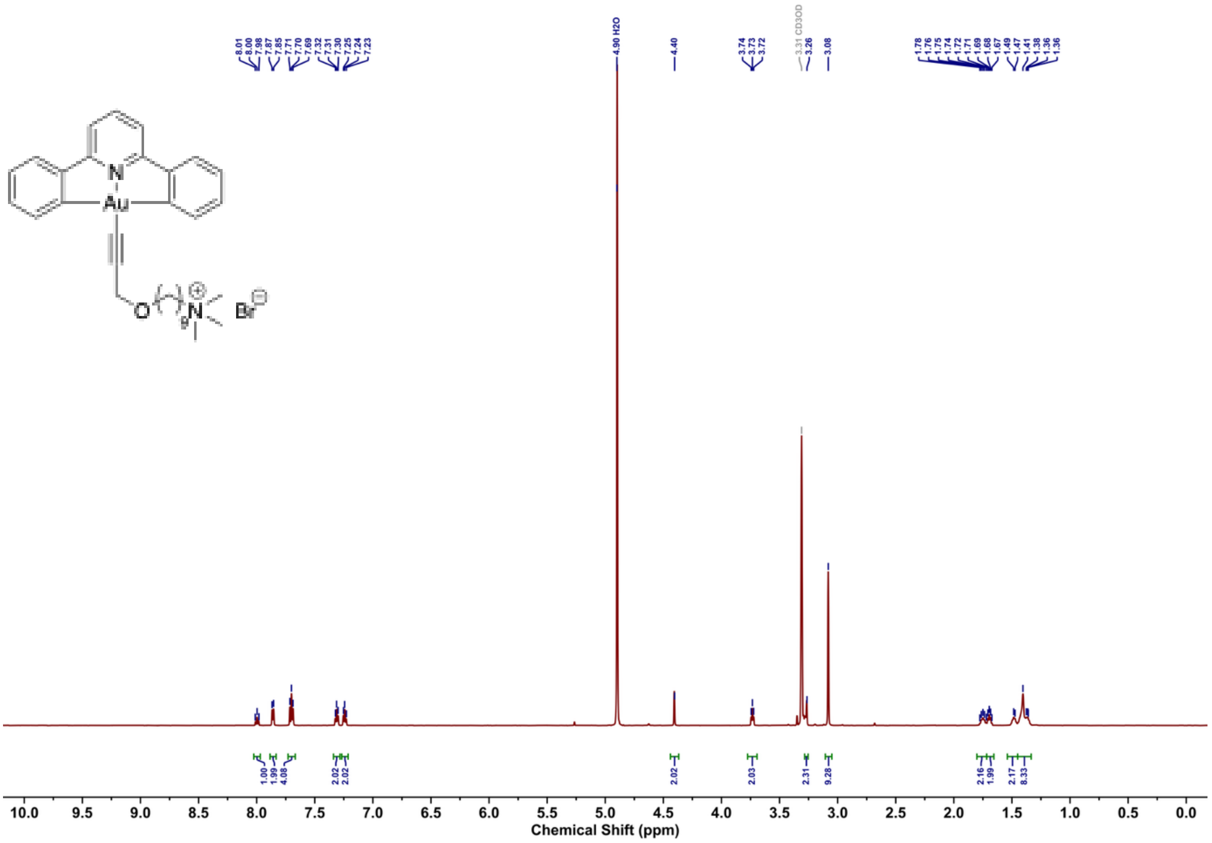

**Figure S12:** $^1$H NMR spectrum (600 MHz) of **GA** in $CD_3OD$ at 20.0 °C.

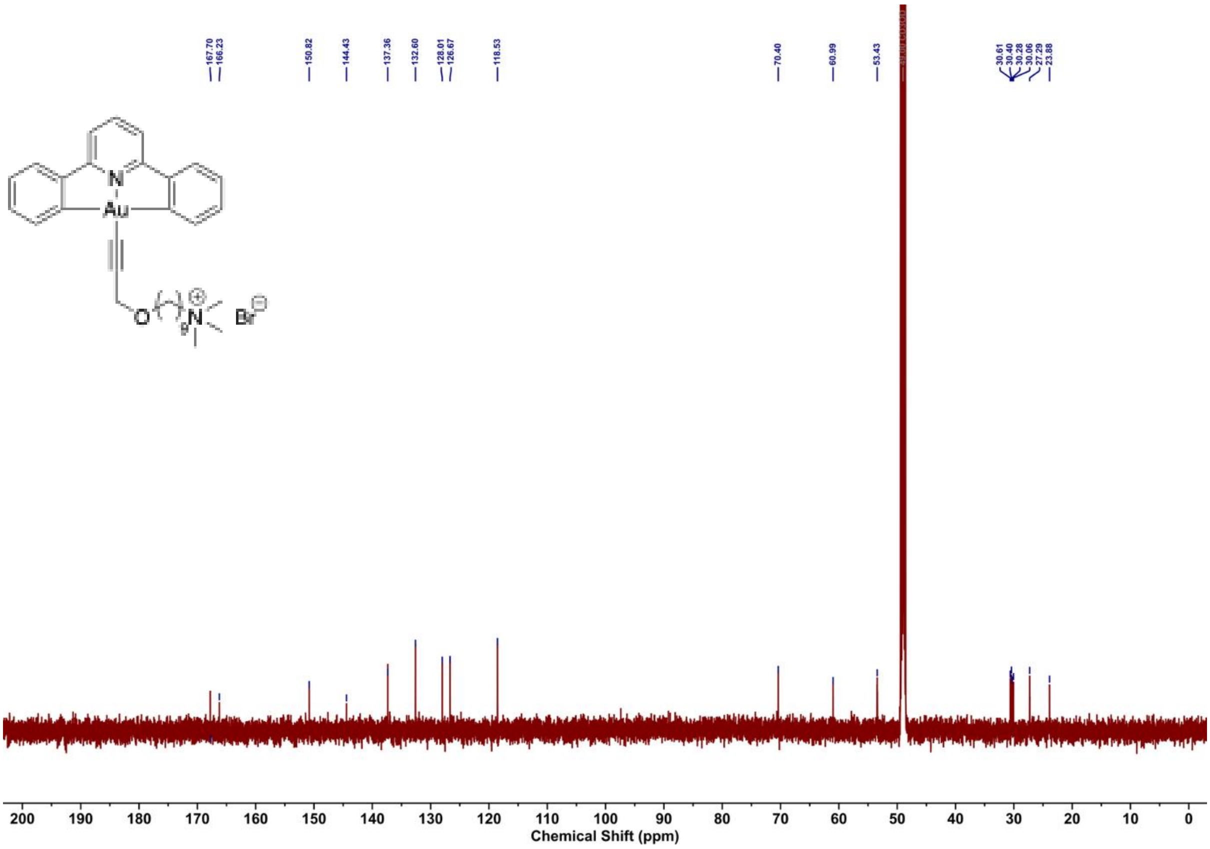


**Figure S13:** $^{13}C$ NMR spectrum (151 MHz) of **GA** in $CD_3OD$ at 20.0 °C.
